# A retrospective study of hypofractionated radiotherapy for keloids in 100 cases

**DOI:** 10.1038/s41598-021-83255-4

**Published:** 2021-02-11

**Authors:** Ping Wen, Taifang Wang, Yueling Zhou, Yue Yu, Chunli Wu

**Affiliations:** Department of Radiation Oncology, the Fourth Affiliated Hospital of China Medical University, Shenyang, 110032 Liaoning China

**Keywords:** Diseases, Medical research

## Abstract

At present, the consensus on the best treatment for keloids is the combination of clinical and surgical therapies, if necessary, associated with adjuvant radiotherapy like brachytherapy. Whereas, the uniform scheme of radiotherapy in keloids is unclear. Here, we conducting a retrospective analysis to assess the efficacy and safety of a specific treatment regimen (20 Gy in 5 fractions) in keloid patients. We retrospectively analysed the medical records of keloid patients receiving auxiliary postoperative radiotherapy (PORT) treatment from 2009 to 2019. The patients were treated with the hypofractionation method of 20 Gy in 5 fractions. We compared the local control rate and complications, using the chi-square test and logistic regression analyses. After screening, we identified 100 keloid patients in this study, with a median follow-up of 59 months. In this study, the overall local control rate of keloid lesions was 84.8%. After multivariate analyses (primary keloid or not, family history, interval from surgery to irradiation and site), our research showed that primary keloid, site and interval from surgery to irradiation were significantly related to recurrence. Acute radiation injury and late radiation injury accounted for 3% (erythema) and 1% (skin sclerosis) of the total cases, respectively. Our results indicate that a postoperative hypofractionation with radiation dose of 20 Gy in 5 fractions may be effective, easy to accept and safe for keloid patients.

## Introduction

Keloid is a benign disease, the mechanism of which is that collagen anabolic function loses its normal restriction and remains in a hyperactive state. It becomes keloid when the growth of cicatrix tissue exceeds the wound to infiltrate into the surrounding normal tissue, or when the growth of cicatrix tissue exceeds the normal growth period. It is characterized by hyperplastic skin fibres^1–3^. Keloids tend to occur in the chest, shoulder, neck, back and ear, which not only affects aesthetics, but also gives rise to local functional obstruction^4^. According to the reason for connective tissue dysplasia, the samples were divided into primary and secondary tissues. Generally speaking, the former usually occurs on the chest or shoulders, and the cause is unknown, while the latter occurs following any skin trauma such as surgery, acne and the most commonly cosmetic piercing^5^. Several risk factors have been proposed, including family history, anatomical site of injury and specific ethnic background. Keloids are known to be more severe in black and Asian people than in white people^6^.

At present, although multiple treatments are available, there is still no consensus on the optimal treatment. Silica gel sheeting (SGS) is a commonly used occlusive dressing to reduce the risk of excessive scar formation. As using SGS requires a high level of patient compliance, as protocols typically require patients to wear SGS for more than 12 h a day and for at least 12 months^7,8^, its widespread use has been limited. Intraregional steroid injection is also a treatment option for many physicians. Triamcinolone can reduce relapse at an average rate of 50% after surgical excision and reduce the volume of the cicatrix. However, it has highly variable response rates to intradermal steroid therapy^9,10^. Surgical excision is the most basic therapeutic method. However, it is still accompanied by a high recurrence rate, which is as high as 50% in 1 year^11^. Currently, postoperative adjuvant radiation therapy has been shown to be an effective method for reducing the rate of keloid relapse, especially brachytherapy^12,13^. Normally, electron irradiation is narrower and the concentration of radioactive deposition, is more suitable for treating local superficial lesions, especially keloids^14^. Radiation therapy can not only weaken immune cell function and the formation of new blood vessels, to strongly inhibit inflammation, but also block the cell cycle and induce premature senescence of cells^15,16^. When patients receive auxiliary postoperative radiotherapy, the recurrence rate depends on the treatment regimen and the location of keloids^17^. Therefore, there are various differences in the reports of radiation dose and fractionation mode. This article investigates the effects and complications of postoperative radiotherapy in keloid patients. The results are expected to provide a new reference for the clinical treatment of keloids.

## Materials and methods

### Patient selection study design

This study was conducted strictly by the STROBE guidelines^18^. From 2009 to 2019, after screening, a total of 100 keloid patients who were treated postoperatively with radiation therapy had sufficient follow-up data for inclusion in this article. The main inclusion criteria were clinical diagnosis with keloids, designated for auxiliary postoperative radiotherapy (within 48 h after surgery) with a total dose of 20 Gy in 5 fractions, and a follow-up time of more than 1 year. All patients' sex age, and keloids type, number, location, symptoms and treatment were included. According to the clinical symptoms and signs of keloids, the Sawada scoring method was used to classify keloids into three levels: mild, moderate and severe^19,20^. Sawada scale is more suitable for Asian, due to the importance attached to the subjective feelings of keloid patients. According to the five factors of redness, elevation, hardness, itching, tenderness and pain of keloids, the keloids were divided into three levels of light, medium and heavy by integral method (each factor was divided into four gradients from mild to severe, ranging from 0 to 3 points). By integral standard, the integral of 11–15 points is severe; 6–10 points is moderate; on a scale of 1–5, it is mild. The follow-up was arranged at 3 months, 6 months, 1 year and every year afterward, which was more than 1 year. In our department, there were 3 radiotherapists responsible for the electron irradiation of skin and benign tumours, including 1 attending physician and 2 resident physicians. The resident physicians were responsible for the follow-up records. All methods were carried out in accordance with relevant guidelines and regulations. The informed consent have been obtained by participant to publish the information and images in an online open-access publication. The ethics committees waived informed consent, and the work was approved by the ethics committees of the Fourth Affiliated Hospital of China Medical University.

### Treatment procedures and radiotherapy

**Figure 1:**
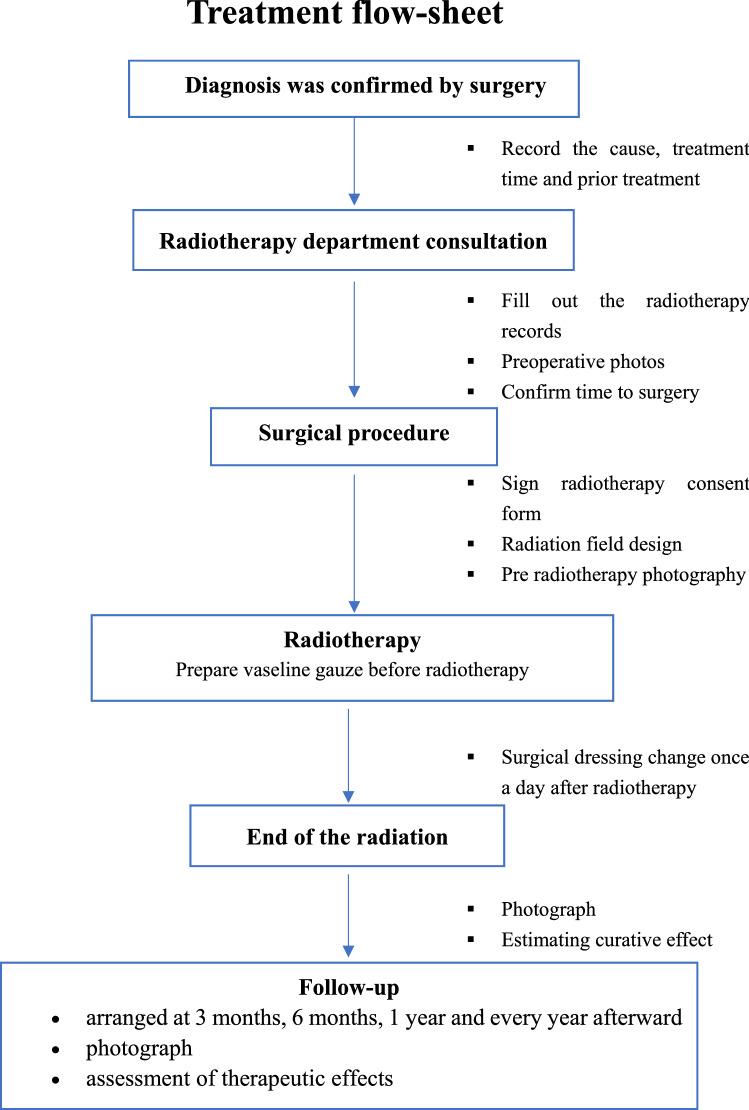
The treatment flow-sheet.

All included patients followed the following treatment process. First of all, the patients were diagnosed as keloid by pathology, then they communicated with the radiotherapist, who would explain the postoperative radiotherapy matters to the patient. Within 48 h after surgery, the patients signed the radiotherapy consent form, and the medical records were taken notes by the radiotherapist. After that, the radiotherapist, dosimetrist and technician then design and perform the radiotherapy. During the therapy, dressing change in the operative area after radiotherapy should be done once a day. At last, on the day of the end of radiotherapy, the radiotherapist evaluated the treatment efficacy of keloid patients and informed the time of follow-up. The treatment flow sheet can be seen in Fig. 1.

Since there is no unified standard dose for PORT for keloids, most scholars believe that the total radiation dose should be controlled within 20 Gy^21,22^. Therefore, all patients were treated with PORT using a linear accelerator with a 6 meV electron source and prescribed the 90% isodose line. Most lesions were treated within 24 h postoperatively (74% within 24 h). The skin surface pad 0.5–1 cm bolus to increase the skin surface dose. The treatment fields radially encompassed the keloid surgical bed with 1.0 cm (the corner extended to 1.5 cm) to surmount the marginal risk. Patients were treated with a total dose of 20 Gy in 5 fractions and received continuous irradiation (except on weekends), with a corresponding biologically equivalent dose (BED) of 28 Gy. As keloids manifested as early-reacting tissue, an α/β ratio of 10 was selected.

### Outcome evaluation and statistical methods

The primary objective of this article was to evaluate the recurrence rate, and keloid recurrence was defined as any sign of keloid at the incision and regardless of the size. Additionally, univariate factor analyses of age, sex, primary status, lesion type, interval from surgery to irradiation, site, family history, Sawada grade and radiotherapy protocol were performed to assess the relationship with local control. In order to control confounding bias, multivariate analysis was used to correctly evaluate the association between exposure factors and keloid. The secondary outcomes were acute and late complications according to the Radiation Therapy Oncology Group (ROTG) grading criteria. Chi squares tests were used for univariate analysis to sieve out the meaningless variables, and multivariate analysis was performed via binary logistic regression models in SPSS program version 22.

## Results

### Patient characteristics

From January 2009 to June 2019, there were 124 keloid patients admitted to our hospital. The aetiology of keloids includes acne, surgery, cosmetic piercing and other primary keloids. Because the follow-up time was not available in 24 cases, 100 patients (with 151 keloid lesions) were included in the study, including 70 females and 30 males, with a median follow-up of 59 months. The median age of the patients was 27 years (8–76 years). Sawada classification for patients with mild-to-moderate disease accounted for 99%. 12 lesions had already undergone one or more other treatments. Of them, 8 lesions had previously been treated with surgery combined with radiotherapy (2 lesions relapsed), and the previous radiation total dose was no more than 16 Gy. One lesion was treated with cortisone infiltration (relapse). One lesion of postsurgical adjuvant radionuclide therapy and 2 lesions of postsurgical combined strontium-89 treatment were included. According to the location of keloids, 82 keloids were located on the face and neck (earlobe keloids accounted for 91.5%) had the best effect, and the local control rate was 92.68. The keloid patients’ characteristics are shown in Table 1.

**Table 1:** Demographic of patients’ keloid characteristics.

**Demographic of patients**	Number of patients (%)
**Sex**	
Male	30 (30)
Female	70 (70)
**Age**	
Median (range)	28 (8–76)
**Follow-up time (M)**	
Mean (range)	59 (12–123)

Characteristics of lesions	Number of lesions (%)
**Lesion type**	
Single	70 (46.4)
Multiple	81 (53.6)
**Primary or not**	
Primary	26 (17.2)
Secondary	125 (82.8)
**Family history**	
Yes	54 (35.8)
No	97 (64.2)
**Site**	
Head and neck	82 (54.3)
Trunk	57 (37.8)
Limbs	12 (7.9)
**Sawada grade**	
Mild	75 (49.7)
Moderate	75 (49.7)
Severe	1 (0.6)
Previous treatment	12 (7.9)

### Treatment efficacy and its influencing factors

**Figure 2:**
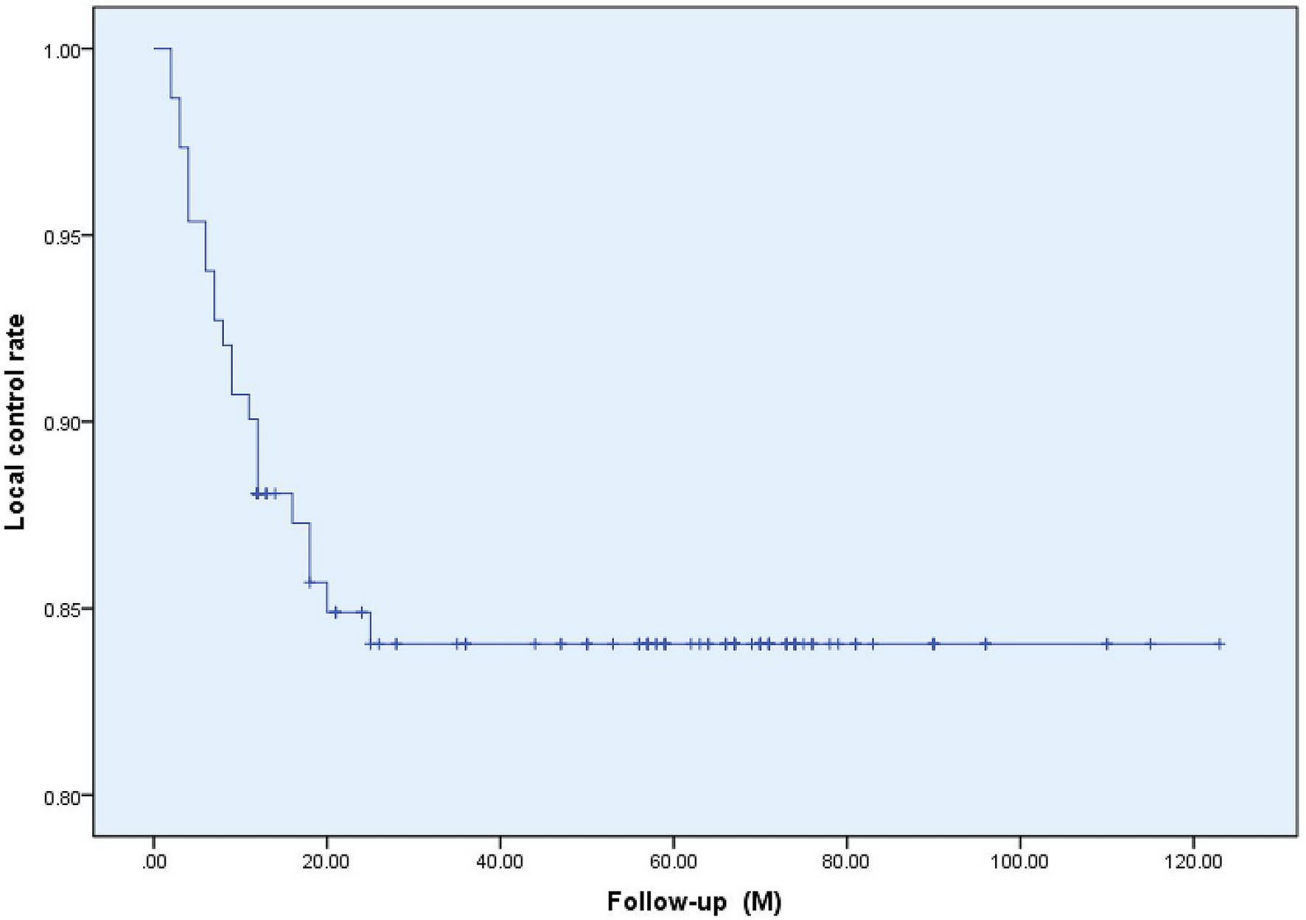
Local control and follow-up of 151 keloids: overall Kaplan–Meier estimated local control to be 84.8%.

**Figure 3:**
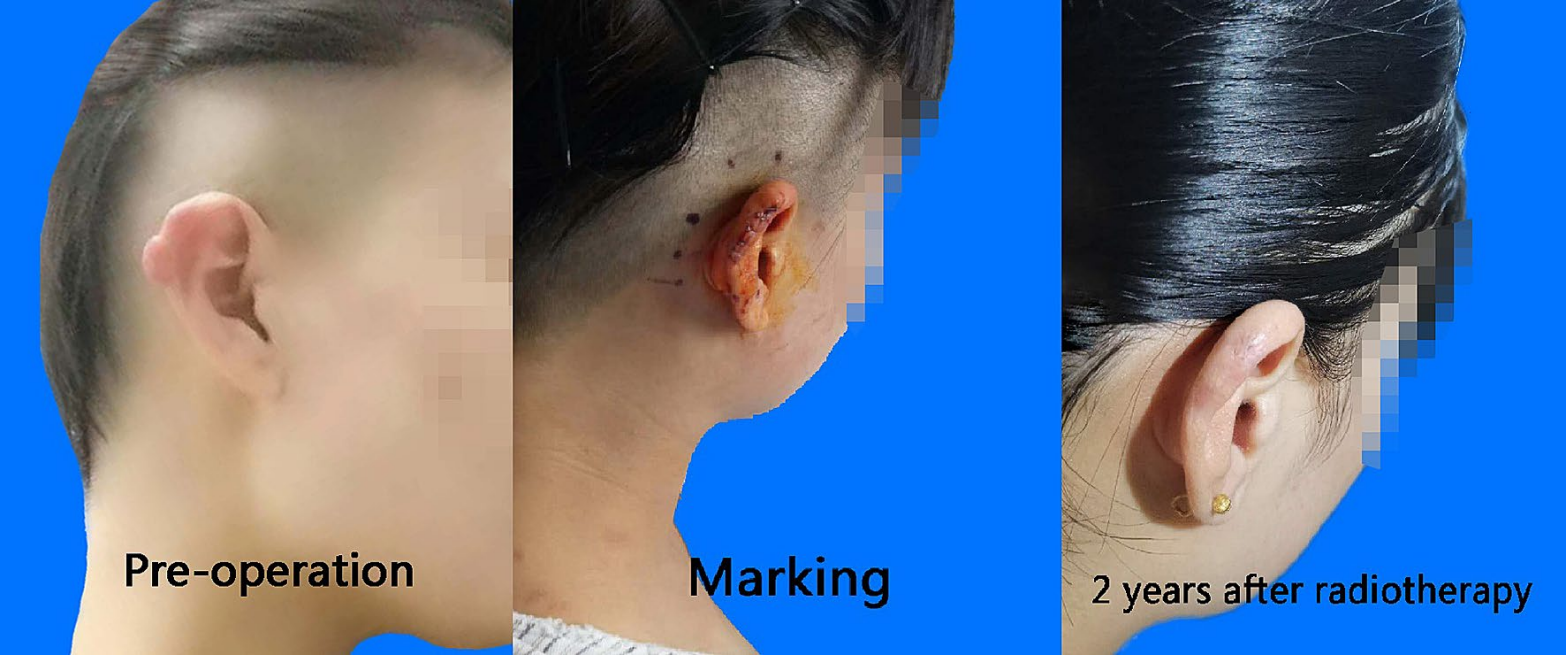
A typical ear keloid.

At the last follow-up date, the effective rate of 151 keloid lesions was 84.8 (accounting for 81 cases). The follow-up states of all keloids as shown in Fig. 2. Fourteen cases of recurrence occurred within 1 year after treatment. Figure 3 shows a good prognosis for ear keloids following up for 24 months. Univariate analysis showed that primary keloids, interval from surgery to irradiation, family history and site were statistically significant in the comparison of all of the above indexes (P < 0.05). More details of the univariate analysis are included in Tables 2. Further multivariate analysis showed that the treatment effect of patients with primary keloids and those receiving PORT with interval times over 24 h showed poor results. The radiotherapy effect of different sites was also statistically significant, among which the head and neck effect was the best, with a local control rate of 92.68. As shown in the Table 3.

**Table 2:** Univariate analysis for keloid recurrence with chi squares.

Variable	Recurrence N = 23(%)	Recurrence-free N = 128(%)	χ^2^ statistic	P value
**Sex**			1.710	0.191
Male	10 (43.5)	38 (29.7)		
Female	13 (56.5)	90 (70.3)		
**Age**			2.132	0.217
< 18	0 (0)	11 (8.6)		
≥ 18	23 (100)	117 (91.4)		
**Lesion type**			1.127	0.288
Single	13 (56.5)	57 (44.5)		
Multiple	10 (43.5)	71 (55.5)		
**Primary or not**			23.259	< 0.001
Primary	12 (52.2)	14 (10.9)		
Secondary	11 (47.8)	114 (89.1)		
**Interval from surgery to irradiation**			7.481	0.006
≤ 24 h	11 (47.8)	97 (75.8)		
> 24 h	12 (52.2)	31 (24.2)		
**Family history**			10.248	0.001
Yes	15 (65.2)	39 (30.5)		
No	8 (34.8)	89 (69.5)		
**Thickness of bolus (cm)**			0.103	0.748
0.5	21 (91.3)	114 (89.1)		
1.0	2 (8.7)	14 (10.9)		
**Site**			9.421	0.009
Head and neck	6 (26.1)	76 (59.4)		
Trunk	15 (65.2)	42 (32.8)		
Limbs	2 (8.7)	10 (7.8)		
**Sawada grade**			1.472	0.479
Mild	9 (39.1)	66 (51.6)		
Moderate	14 (60.9)	61 (47.7)		
Severe	0 (0)	1 (0.7)

**Table 3:** Multivariate analysis for keloid recurrence with a linear regression model.

Variable	N (%)	Recurrence rate (%)	P value
**Primary keloid**			< 0.001
Yes	26 (17%)	46.20	
No	125 (83%)	8.80	
**Family history**			0.064
Yes	54 (36%)	25.93	
No	97 (64%)	9.28	
**Interval from surgery to irradiation**			0.002
≤ 24 h	108 (72%)	10.19	
> 24 h	43 (28%)	27.91	
**Site**			0.018
Head and neck	82 (54%)	7.31	
Trunk	57 (38%)	26.32	
Limbs	12 (8%)	16.67	

### Complications: adverse events

In all of the follow-up cases, erythema as an early adverse event of the radiation area occurred in 3 cases. One case of late radiation injury with earlobe skin sclerosis occurred. All the above was regarded as skin reaction level 1 according to the standard for the RTOG grading scale. Moreover, no secondary malignancies associated with radiotherapy were found during the follow-up.

## Discussion

The aim of keloid treatment is to remove symptoms and improve the cosmetic aspect of the cicatrix without any recurrence. Thereout, a variety of keloid treatments are derived. PORT, as one of the treatments, has been used for nearly a century, and excellent therapeutic effects have been achieved^23^. Research shows that fibroblasts are the major repair cells in wound healing, and dysfunction can lead to keloids^24,25^. High-dose-rate (HDR) brachytherapy can damage the DNA strand of hyperactive scar cells, and inhibiting angiogenesis in keloid formation by affecting local lymphocytes and macrophages blocks the inflammatory process, thus delaying scar growth. As several studies have presented, depending on the body part and BED radiotherapy, the control rates of keloids are approximately 70–90%^26–31^. According to the latest non-malignant disorder technology and evidence-based practice guidelines issued by Germany in 2015, a total dose of 16–20 Gy within 24 h of resection and in 5 fractions within 1 week are recommended, and the results range from 60 to 80%^32^.

In this study, hypofractionated radiotherapy with 20 Gy was divided 5 times, which was converted into the equivalent biological dose of 28 Gy. The local control rate is 84.8%, with a low incidence of adverse reactions. In general, carcinogenesis by radiotherapy has emerged in a few years to decades. The carcinogenic effect was related to the exposure site, patient sex, age, susceptibility gene and dose rate of radiation^33^. In this study, the longest follow-up time was 123 months, and no cases of secondary malignancies were found.

More inclined to receive early postoperative irradiation domestically and abroad. Since trauma days 1–3 is the most active period of fibroblast proliferation, early radiotherapy is the best period to intervene in scar formation. Within 24 h after surgery, immature fibroblasts grew in most incisions, of which unstable collagen fibres were the main components. These cells were sensitive to radiation, and radiation could effectively inhibit the proliferation of immature fibroblasts^34,35^. Postoperatively, according to the results of this study, there was an obvious correlation between the time and the efficacy of radiotherapy. The irradiation time began 24 h, postoperatively, and the results were better. There is also a correlation between the cause of scar formation and the therapeutic effect. Our study showed that primary keloids were less effective than secondary keloids. The formation of keloids is influenced by many factors, possibly combining multiple genes and external factors. However, it is of great significance to study the related pathogenic genes to reveal the difference in mechanism. In this study, the treatment effect of the earlobe keloid was significantly better than site of trunk and limbs, and patients' response were better too. Consider the cicatrix area of the ear more small, and for tension-free skin, which was consistent with the research result of Ogawa et al.^36,37^. To better balance local control and adverse reactions, BED postoperative radiotherapy may be refined according to the cicatrix area.

There are still some limitations in our study. Several cases were followed up by phone, which may lead to underestimation of the complications of PORT, not to mention their cosmetic effects. Second, whereas epidemiological surveys show that people of colour, such as blacks and Asians, suffer from keloids, the incidence rate of keloids is significantly higher than that in Caucasians^38,39^. Since all the subjects included in our study were Asians, the data results were inevitably biased. Third, the lack of a control group is another limitation. Given the limitations of our study, more large scale, better designed studies should be performed to validate our conclusions.

## References

[1] Marneros AG, Krieg T (2004). Keloids–clinical diagnosis, pathogenesis, and treatment options. J. Dtsch. Dermatol. Ges..

[2] Bock O, Schmid-Ott G, Malewski P, Mrowietz U (2006). Quality of life of patients with keloid and hypertrophic scarring. Arch. Dermatol. Res..

[3] Gauglitz GG, Korting HC, Pavicic T, Ruzicka T, Jeschke MG (2011). Hypertrophic scarring and keloids: Pathomechanisms and current and emerging treatment strategies. Mol. Med..

[4] Carvajal CC, Ibarra CM, Arbulo DL, Russo MN, Solé CP (2016). Postoperative radiotherapy in the management of keloids. Ecancermedicalscience.

[5] Arneja JS, Singh GB, Dolynchuk KN, Murray KA, Rozzelle AA, Jones KD (2008). Treatment of recurrent earlobe keloids with surgery and high-dose-rate brachytherapy. Plast. Reconstr. Surg..

[6] Ogawa R (2017). Keloid and hypertrophic scars are the result of chronic inflammation in the reticular dermis. Int. J. Mol. Sci..

[7] Gold MH, McGuire M, Mustoe TA, Pusic A, Sachdev M, Waibel J, Murcia C, Management, I. A. P. o. S (2014). Updated international clinical recommendations on scar management: Part 2–algorithms for scar prevention and treatment. Dermatol. Surg..

[8] Heppt MV, Breuninger H, Reinholz M, Feller-Heppt G, Ruzicka T, Gauglitz GG (2015). Current strategies in the treatment of scars and keloids. Facial Plast. Surg..

[9] Trisliana Perdanasari A (2014). Recent developments in the use of intralesional injections keloid treatment. Arch. Plast. Surg..

[10] Muneuchi G, Suzuki S, Onodera M, Ito O, Hata Y, Igawa HH (2006). Long-term outcome of intralesional injection of triamcinolone acetonide for the treatment of keloid scars in Asian patients. Scand. J. Plast. Reconstr. Surg. Hand Surg..

[11] Furtado F, Hochman B, Ferreira LM (2012). Evaluating keloid recurrence after surgical excision with prospective longitudinal scar assessment scales. J. Plast. Reconstr. Aesthet. Surg..

[12] Guix B, Henríquez I, Andrés A, Finestres F, Tello JI, Martínez A (2001). Treatment of keloids by high-dose-rate brachytherapy: A seven-year study. Int. J. Radiat. Oncol. Biol. Phys..

[13] Mankowski P, Kanevsky J, Tomlinson J, Dyachenko A, Luc M (2017). Optimizing radiotherapy for keloids: A meta-analysis systematic review comparing recurrence rates between different radiation modalities. Ann. Plast. Surg..

[14] Xu J, Yang E, Yu NZ, Long X (2017). Radiation therapy in keloids treatment: History, strategy, effectiveness, and complication. Chin. Med. J. (Engl.).

[15] Ogawa R (2019). Diagnosis and treatment of keloids and hypertrophic scars-Japan Scar Workshop Consensus Document 2018. Burns Trauma.

[16] Ji J (2015). Ionizing irradiation inhibits keloid fibroblast cell proliferation and induces premature cellular senescence. J. Dermatol..

[17] Gold MH, Nestor MS, Berman B, Goldberg D (2020). Assessing keloid recurrence following surgical excision and radiation. Burns Trauma.

[18] Cuschieri S (2019). The STROBE guidelines. Saudi J. Anaesth..

[19] Sawada Y, Sone K (1992). Hydration and occlusion treatment for hypertrophic scars and keloids. Br. J. Plast. Surg..

[20] Li H, Cai J, Liu Z, Zhao J (2004). Evaluation of the application of comprehensive criteria in the diagnosis and treatment of keloid. Chin. J. Aesth. Med..

[21] Bischof M, Krempien R, Debus J, Treiber M (2007). Postoperative electron beam radiotherapy for keloids: Objective findings and patient satisfaction in self-assessment. Int. J. Dermatol..

[22] Aköz T, Gideroğlu K, Akan M (2002). Combination of different techniques for the treatment of earlobe keloids. Aesthetic Plast Surg.

[23] van Leeuwen (2015). Surgical excision with adjuvant irradiation for treatment of keloid scars: A systematic review. Plast. Reconstr. Surg. Glob. Open.

[24] Shen J (2015). Hypofractionated electron-beam radiation therapy for keloids: Retrospective study of 568 cases with 834 lesions. J. Radiat. Res..

[25] Dong X, Mao S, Wen H (2013). Upregulation of proinflammatory genes in skin lesions may be the cause of keloid formation (Review). Biomed. Rep..

[26] Darzi MA, Chowdri NA, Kaul SK, Khan M (1992). Evaluation of various methods of treating keloids and hypertrophic scars: A 10-year follow-up study. Br. J. Plast. Surg..

[27] Sclafani AP, Gordon L, Chadha M, Romo T (1996). Prevention of earlobe keloid recurrence with postoperative corticosteroid injections versus radiation therapy: A randomized, prospective study and review of the literature. Dermatol. Surg..

[28] Emad M, Omidvari S, Dastgheib L, Mortazavi A, Ghaem H (2010). Surgical excision and immediate postoperative radiotherapy versus cryotherapy and intralesional steroids in the management of keloids: A prospective clinical trial. Med. Princ. Pract..

[29] Perez CA, Lockett MA, Young G (2001). Radiation therapy for keloids and plantar warts. Front. Radiat. Ther. Oncol..

[30] Ragoowansi R, Cornes PG, Moss AL, Glees JP (2003). Treatment of keloids by surgical excision and immediate postoperative single-fraction radiotherapy. Plast. Reconstr. Surg..

[31] Kutzner J, Schneider L, Seegenschmiedt MH (2003). Radiotherapy of keloids. Patterns of care study—results. Strahlenther. Onkol..

[32] Seegenschmiedt MH, Micke O, Muecke R, (GCG-BD), G. C. G. o. R. f. N.-m. D (2015). Radiotherapy for non-malignant disorders: State of the art and update of the evidence-based practice guidelines. Br. J. Radiol..

[33] Hendry JH (2001). Genomic instability: Potential contributions to tumour and normal tissue response, and second tumours, after radiotherapy. Radiother. Oncol..

[34] Hoang D, Reznik R, Orgel M, Li Q, Mirhadi A, Kulber DA (2017). Surgical excision and adjuvant brachytherapy vs external beam radiation for the effective treatment of keloids: 10-year institutional retrospective analysis. Aesthet. Surg. J..

[35] Rödel F (2017). Basics of radiation biology when treating hyperproliferative benign diseases. Front Immunol..

[36] Ogawa R, Mitsuhashi K, Hyakusoku H, Miyashita T (2003). Postoperative electron-beam irradiation therapy for keloids and hypertrophic scars: Retrospective study of 147 cases followed for more than 18 months. Plast. Reconstr. Surg..

[37] Ogawa R (2007). Postoperative radiation protocol for keloids and hypertrophic scars: Statistical analysis of 370 sites followed for over 18 months. Ann. Plast. Surg..

[38] Kouotou EA (2019). Epidemiology and clinical features of keloids in Black Africans: A nested case–control study from Yaoundé, Cameroon. Int. J. Dermatol..

[39] Koonin AJ (1964). The aetiology of keloids: A review of the literature and a new hypothesis. S. Afr. Med. J..

